# Computational analysis of the mesenchymal signature landscape in gliomas

**DOI:** 10.1186/s12920-017-0252-7

**Published:** 2017-03-09

**Authors:** Orieta Celiku, Anita Tandle, Joon-Yong Chung, Stephen M. Hewitt, Kevin Camphausen, Uma Shankavaram

**Affiliations:** 1 Radiation Oncology Branch, Center for Cancer Research, National Cancer Institute, National Institutes of Health, Bethesda, 10 Center Drive, Bldg. 10, Rm. B3B70, Bethesda, MD 20892 USA; 2 Experimental Pathology Laboratory, Laboratory of Pathology, Center for Cancer Research, National Cancer Institute, National Institutes of Health, Bethesda, MD 20892 USA

**Keywords:** Glioma, Computational modeling, Epithelial to mesenchymal transition, CD44

## Abstract

**Background:**

Epithelial to mesenchymal transition, and mimicking processes, contribute to cancer invasion and metastasis, and are known to be responsible for resistance to various therapeutic agents in many cancers. While a number of studies have proposed molecular signatures that characterize the spectrum of such transition, more work is needed to understand how the mesenchymal signature (MS) is regulated in non-epithelial cancers like gliomas, to identify markers with the most prognostic significance, and potential for therapeutic targeting.

**Results:**

Computational analysis of 275 glioma samples from “The Cancer Genome Atlas” was used to identify the regulatory changes between low grade gliomas with little expression of MS, and high grade glioblastomas with high expression of MS. TF (transcription factor)-gene regulatory networks were constructed for each of the cohorts, and 5 major pathways and 118 transcription factors were identified as involved in the differential regulation of the networks. The most significant pathway - Extracellular matrix organization - was further analyzed for prognostic relevance. A 20-gene signature was identified as having prognostic significance (HR (hazard ratio) 3.2, 95% CI (confidence interval) = 1.53–8.33), after controlling for known prognostic factors (age, and glioma grade). The signature’s significance was validated in an independent data set. The putative stem cell marker CD44 was biologically validated in glioma cell lines and brain tissue samples.

**Conclusions:**

Our results suggest that the differences between low grade gliomas and high grade glioblastoma are associated with differential expression of the signature genes, raising the possibility that targeting these genes might prolong survival in glioma patients.

**Electronic supplementary material:**

The online version of this article (doi:10.1186/s12920-017-0252-7) contains supplementary material, which is available to authorized users.

## Background

Gliomas are tumors of the central nervous system and are classified based on histologic type and malignancy grade. Most diffuse gliomas are classified into four histological grades: Grades I and II (low grade), III (anaplastic), or IV (glioblastoma) [1]. Among gliomas, low grade gliomas are typically the least aggressive, with longer, indolent clinical course; glioblastomas (GBMs) on the other hand are highly aggressive cancers with a median survival of 12–18 months’ post diagnosis [2]. The differences in clinical course suggest that low grade gliomas have distinctive genetic features and molecular pathogenesis relative to high grade gliomas [3]. Until recently, histologic diagnosis was the gold standard for the classification of gliomas, forming the basis for clinical diagnosis, prognosis and treatment management [4]. Histologic diagnosis is problematic for cases with mixed histology; worse, it cannot account for the vast molecular heterogeneity found in gliomas. Recent identification of molecular biomarkers for different subsets of gliomas has enabled introduction of molecular characteristics, alongside histopathological features, in the definition and diagnosis of gliomas [5]. These molecular characteristics include: genetic profiling, proteomics, epigenetic changes, and molecular signatures of processes such as epithelial to mesenchymal transition (EMT) [6–10].

Processes that mimic EMT, a normal development process, are often adapted by cancer cells to remodel the extracellular matrix when invading and metastasizing [11]. Such processes are also thought to confer resistance to radiotherapy and chemotherapy [12, 13]. The factors that induce EMT in other cancers may also activate mesenchymal features in gliomas. Furthermore, EMT is an important inducer of the cancer stem cell phenotype [14]. The mesenchymal subtype of glioblastoma typically expresses neural stem cell markers and is associated with an aggressive phenotype [6, 15, 16]. Glioma cells that express stem cell markers are highly invasive and resistant to chemotherapy and radiotherapy in vitro [17–19] and in the clinical setting [20]. Therefore, a better understanding of the molecular mechanism of mesenchymal signature (MS) may help identify and rationally design therapeutic regimes for patient subgroups that may benefit from additional treatments [21].

Progress has been made through in vitro and in vivo animal models in understanding the molecular processes of EMT. However, the dynamics between tumor cells, stem cells, and tumor microenvironment are complex and nonlinear. In the context of non-epithelial tumors, like gliomas, the manifestation of mesenchymal transition is even less understood. Computational modeling and bioinformatics approaches are increasingly proving helpful in obtaining biological insight in the field of oncology [22]. Recently, Cheng et al. evaluated a 64-gene MS signature, previously identified as a marker of aggression and invasiveness in a multi-cancer computational analysis, and found that its expression was associated with prolonged time to recurrence in GBMs [23, 24]. In another report, a 16-gene signature was shown to distinguish anaplastic astrocytoma from GBM [25].

In this study, we hypothesized that high grade gliomas (HGG) (Grade IV) express a distinct mesenchymal signature (MS) compared to low grade gliomas (LGG) (Grade I + II), and investigated the transcription-factor regulation of the signature. Using a combination of computational modeling and bioinformatics approaches, we analyzed 151 primary untreated GBM samples, and 124 primary untreated Grade II LGG samples from The Cancer Genome Atlas (TCGA) to extract the MS, and transcription factors involved in its regulation. Here, we report our findings from the TCGA data. We validated our results in silico in an independent dataset. We also biologically validated CD44 - one of the signature genes - in GBM tumor cells and brain tissue samples.

## Methods

Our approach to the analysis consists of the following primary steps with input and output results shown in parenthesis:

1. Selection of cohorts with low and high expression of MS (In: 275 samples; out: 138 samples).
2. Extraction of key differentially regulated genes, transcription factor networks, and pathways enrichment analysis between the low and high MS cohorts (In: 20500 genes; out: 57 genes)
3. Extraction of a prognostic signature among the key differentially regulated genes (In: 57 genes; out: 20 genes).
4. Validation of the prognostic power of the signature with independent data (276 samples).
5. Validation of biological features of one of the signature genes in vitro (2 GBM cells; 3 Stem cells) and glioma patient tissue samples (57 samples and 4 normal control samples).

The rest of this section elaborates on these steps.

### Selection of cohorts with low and high expression of MS

RNASeqV2 Level-3 gene expression data (MapSplice and RSEM computed) and clinical data were downloaded with TCGA Assembler [26] using build of 06/06/2014. We selected 275 samples, with 151 primary untreated Grade IV GBM samples and 124 primary untreated Grade II LGG samples (40 astrocytomas, and 84 oligodendrogliomas) in the analysis. The expression data were normalized using variance stabilized transformation with R’s *DESeq2* package.

Molecular classification and characteristics of the samples were downloaded from the TCGA landscape publications, Brennan et al. [9], and Brat et al. [27]. A summary of clinical and molecular characteristics is shown in Table 1.

**Table 1:** Clinical data summary

	GBM (Grade IV)		LGG (Grade II)	
	Total	High MS	Total	Low MS
Cases	151	69	124	69
Histological Type				
Primary GBM	151 (100%)	69 (100%)		
Astrocytoma			40 (32.3%)	19 (31.9%)
Oligodendroglioma			84 (67.7%)	50 (68.1%)
Age at Diagnosis (Years)				
Mean	60.8	62.2	41.6	41
Gender				
Female	54 (35.8%)	27 (39.1%)	57 (46%)	35 (50.7%)
Male	97 (64.2%)	42 (60.9%)	67 (54%)	34 (49.3%)
Vital Status				
Alive	52 (34.4%)	22 (31.9%)	111 (89.5%)	67 (97.1%)
Deceased	98 (64.9%)	47 (68.1%)	13 (10.5%)	2 (2.9%)
< NA>	1 (0.7%)			
Survival (Days)				
Mean	338	307	800	754

Our starting point was a MS of 64 genes extracted in a multi-cancer setting and associated with reduced time to recurrence in GBM [23, 24]. The gene expression values for the 64-gene profile for each sample were averaged to form a single value for each tumor, termed a metagene score, as described in Cheng et al. [24]. Each of the 275 (GBM + LGG) samples were ordered according to the metagene score, with higher metagene scores representing samples with higher degrees of transition into the mesenchymal phenotype and, therefore, expected to have more aggressive phenotypes and more unfavorable outcomes. Quantile based selection resulted in 138 samples, with 69 samples in each cohort with 75% high/unfavorable metagene scores designated as high MS vs 25% low/favorable score designated as low MS.

### Extraction of key differentially genes between low and high MS cohorts

#### Differential gene expression

Differential gene expression between the High vs. Low MS cohorts was determined using R’s *DESeq2* package. Five thousand six hundred sixty genes with |log2FC| ≥1 and FDR adjusted *p*-value ≤ 0.05 were selected for construction of regulatory networks.

#### Construction of transcription-factor regulatory networks

Passing messages between biological networks to refine predicted interactions (PANDA) [28] method was used to construct and compare (transcription factor) regulatory networks for the Low and High MT cohorts. The inputs to PANDA are two gene expression matrices, Transcription factor (TF)-gene motif data, and Protein-Protein Interaction (PPI) data; the last is optional. The Low and High MS expression data for the 5660 differentially expressed genes were used as expression matrices. The TF-gene motif data were generated using HAYSTACK software [29]. PANDA integrates expression data, TF-gene motif data, and PPI data to estimate biological networks where “effector” TFs can, through interactions with promoter regions, influence the expression of the “affected” gene targets. The estimated networks are represented in terms of TF-target edges weighted according to the agreement between the different data types - the weights can be positive, measuring the confidence in the existence of an edge in a network, but also negative, measuring the confidence in the lack of existence of an edge in a network.

We used PANDA with leave-1-out Jacknifing approach following Glass et al. [30]. For each of the conditions (Low/High MS), PANDA was run 69 times on expression matrices where one of the samples was left out to generate an ensemble of 69 regulatory networks. Each ensemble of networks was aggregated into a single network, by averaging the weights of the network edges. The edges were also assigned FDR (false discovery rate) -adjusted *p*-values by running t-tests between the weights of the edge in each of the condition. The networks were filtered by keeping edges that had a difference in weight of at least 1 between the two conditions, and an adjusted -log10 FDR of larger than 25; these parameters were chosen based on the distribution of the edge weights and -log10 FDRs. The resulting networks were analyzed by PANDA to summarize which subnetworks were uniquely regulated in each of the conditions.

Following Glass et al. [30] we also set out to compare the networks’ characteristics with networks generated by randomly permuting the gene labels of one, or both gene expression matrices. This resulted in a decrease in the size of the networks satisfying the mentioned edge-filtering criteria. Permutation of the gene labels for the High MS gene expression matrix had a larger effect in the reduction of the network. Permutation of the gene labels of both gene expression matrices resulted in no edges satisfying the criteria. PANDA also enables comparison of networks to determine which genes are differentially regulated between the networks, in the sense that the *indegree* - the number of regulatory edges going into a gene - is significantly different in one of the networks versus the others. Genes with indegree changes (1157), with *p*-value < = 0.05 were considered differentially regulated between the two networks.

#### Pathway enrichment of differentially regulated genes

R’s *reactomePA* library was used to perform pathway enrichment of 1157 differentially regulated genes. Extracellular Matrix Organization (EMO) is one of the top five pathways enriched with differentially regulated genes. We focused on the set of EMO genes (35 genes), expanded with the TFs regulating them (22 genes) - these are TFs connected by edges in the regulatory network of one or both phenotypes, a total of 57 genes were further analyzed for prognostic significance.

### Extraction of a prognostic signature among the key differentially regulated genes

LASSO [31] is a penalized regression method suited for constructing models with potentially large number of covariates, and can be used even when the number of covariates exceeds the number of samples. LASSO implemented in R’s *penalized* package was used to perform Cox Proportional Hazards Regression on the EMO genes. *Age* and *type* (LGG versus GBM) were also used as covariates in the model, since they are known prognostic markers. The output of LASSO regression is a set of 20 genes with non-zero coefficients, which we further call LASSO-prioritized genes.

We evaluated the combined prognostic power of the LASSO-prioritized genes using a “prognostic index” (PI) approach [32]. PI, also known as the risk score, is computed as the linear component of the Cox model, $PI = {\hat{\beta}}_1{x}_1+{\hat{\beta}}_2{x}_2+ \dots +{\hat{\beta}}_n{x}_n$ where *x*_*i*_ is the expression value of the i-th gene and ${\hat{\beta}}_i$ is the corresponding coefficient from the Cox fitting. The fitting was performed using R’s *survival* package. The PI scores were used to determine risk groups, by stratifying the samples down the median of the PI value (higher values for higher risk). For the resulting two groups, a log-rank test was performed.

### Validation of the prognostic power of the signature with independent data

GSE16011 data set was used for independent analysis. The data set consists of Affymetrix GeneChip Human Genome U133 Plus 2.0 Array data for 276 glioma samples with Grade I-IV, and 8 (normal adult brain) control samples. R’s *GEOquery* package was used to download the data from the Gene Expression Omnibus (GEO). A summary of clinical characteristics is shown in Additional file 1: Table S1. To show validation the of 20 gene LASSO prioritized genes in the validation data, we performed unsupervised hierarchical clustering, PCA (principal component analysis) (with R’s *made4* package), and multi-gene Cox-Proportional Hazards analysis.

### Validation of biological features of one of the signature genes

#### Cell lines

The U-87 MG, LN18 (ATCC, Manassas, VA) and the U251 (National Cancer Institute Frederick Tumor Repository) human GBM cell lines were grown in Dulbecco’s Modified Eagle Medium (DMEM) (Invitrogen, Carlsbad, CA) with 10% fetal bovine serum (FBS), and maintained at 37 °C, 5% CO2. Cells were authenticated by the supplier using STR profiling, isoenzyme analysis, karyotype analysis, morphologic analysis, contamination testing and were used within 6 months.

U251-GFP cells were created by transducing with lentiviral particles expressing green fluorescent protein (sc-108084, Santa Cruz) and selecting using puromycin dihydrochloride.

Four neurosphere-forming cells were isolated from human GBM surgical specimens: GBMJ1 and GBAM1; NSC2326 (kindly provided by Dr. Frederick Lang, MD Anderson Cancer Center), and 0923 (kindly provided by Dr. Howard Fine, NCI, NIH). Neurospheres were maintained in stem cell medium consisting of DMEM/F-12 (Invitrogen), B27 supplement (1X) (Invitrogen), and human recombinant bFGF and EGF (50 ng/mL each) (Sigma). All cultures were maintained at 37 °C in an atmosphere of 5% CO2.

#### siRNA-based transfection

CD44 gene is a well-known marker and shown to express in many cancers to play a significant role in the MS phenotype. To test the role of CD44 inhibition, we used gene specific siRNAs. For siRNA transfections, 2-pmol of either siCD44_1 (5′-CTGAAATTAGGGCCCAATTAA-3′, SI00012775) or siCD44_5 (5′-AACTCCATCTGTGCAGCAAAC -3′, S100299705) (Qiagen Inc., Germantown, MD) was complexed with RNAi Max lipid transfection reagent (Invitrogen) in DMEM media for 15 min at ambient temperature. Two thousand cells suspended in DMEM supplemented with 20% FBS were then added. Plates were maintained at ambient temperature for 15 min before being placed at 37 °C/5% CO2. Cell viability was assessed 24 and 48 h post siRNA transfection through quantification of ATP (CellTiter-Glo luminescent Reagent, Promega, Madison, WI). Untransfected cells and wells transfected with negative (All star siNegative [siNeg], Qiagen) and positive (All star siCelldeath, Qiagen) control siRNAs were used as controls. Protein for Western blot analysis was harvested 6 or 24 h post siRNA transfection.

#### Western blot analysis

Gene expression was analyzed using western blot analysis. Cell pellets were lysed on ice in RIPA buffer (Pierce, Rockford, IL) supplemented with Complete Mini EDTA-free Protease Inhibitor Cocktail (Roche, Indianapolis, IN) and Phosphatase Inhibitor Cocktail (Sigma, St. Louis, MO). Protein concentrations were determined by Bradford assay (Bio-Rad, Hercules, CA). Protein (40 μg) was diluted 1:5 in 5X protein loading buffer (Fermentas, Glen Burnie, MD), boiled at 80 °C for 5 min, electrophoresed on a 4–20% Tris-Glycine gel, and transferred using a Trans-Blot Turbo Transfer System (Bio-Rad, Hercules, CA). Membranes were blocked in 5% Non-fat milk powder (BioRad), incubated with primary antibody overnight at 4 °C, incubated with HRP-coupled secondary antibody 1 h at room temperature, developed with Visualizer Western Blot Detection Kit (Millipore, Billerica, MA), and visualized on a LAS-4000 imager (Fujifilm, Edison, NJ). The following antibodies were used at 1: 1000 dilutions: human anti-CD44 (#3570S, Cell Signaling Technology) and mouse anti-actin (MAB 1501R, Millipore). Secondary antibody, anti-mouse-HRP (Santa Cruz Biotechnology, Santa Cruz, CA) was used at 1:10,000 dilutions.

#### Scratch assay for migration

To study the role of CD44 in cellular migration, scratch assay was used. U251 GFP cells were transfected in 10 cm dishes. Twenty-four hours post-transfection, cells trypsinized and were seeded into 24-well cell culture plates to create a confluent monolayer. Reference marking was created by scratching the outer surface of the well with a needle. Using a p200 pipet tip a scratch was made perpendicular to the reference mark onto the cell monolayer. Wells were washed twice with PBS and replaced with the desired medium. Fluorescence images were obtained for the same scratched region until the scratch closed completely.

#### Boyden chamber assay for invasion

The role of CD44 in cellular invasion was assessed using the Boyden chamber assay. U251 GFP cells were transfected in 10 cm dishes. Twenty-four hours post-transfection, cells trypsinized and were used for invasion assay using the matrigel coated Boyden chambers (#354480, BD Biosciences). U251GFP cells either transfected with siNeg, or siCD44 were placed in the upper well of a Boyden migration chamber which is separated from the lower well by a porous filter coated with basement membrane matrix, matrigel. Media containing 1% FBS was placed in the lower well as a chemoattractant to facilitate cell invasion. After 24-h incubation, cells from the top surface were scraped off, membranes were detached, and cells invaded to the bottom surface were photographed (In Cell Analyzer 2000, GE Healthcare Life Sciences). The fluorescent intensity was measured using the Synergy/H1 microplate reader (BioTek, Vermont).

For GBM stem cell invasion assay; NSC11 stem cells as single cells were placed in the upper well of a Boyden migration chamber. The lower chamber was seeded with U251 cells either transfected with siNeg, or siCD44. DMEM media containing 1% FBS was used as a chemoattractant to facilitate stem cell invasion.

#### Clonogenic survival assay

The clonogenic potential of cells with downregulated CD44 expresson was analyzed using the clonogenic survival assay. Cells were transfected in 10 cm dishes. Twenty-four hours post-transfection, cells trypsinized and were seeded into six-well tissue culture plates. Twelve days after seeding, colonies were stained with crystal violet. The number of colonies containing at least 50 cells was determined and the surviving fractions were calculated.

#### Immunohistochemistry

To test the clinical correlation of CD44 expression, we used immunohistochemical analysis in glioma patient tumor samples. Tissue microarray (TMA) slides were purchased from US Biomax, Inc. (Cat. # BS17017a, Rockville, MD). There were a total of 30 cases of astrocytoma (20 Grade I-II, 10 Grade III), 27 glioblastoma (GBM), and 4 adjacent normal brain tissues. One case among astrocytoma specimens was mixed with grade 4 and considered subsequently as a GBM case in the analysis. The TMA sections were baked at 60 °C for 30 min and then deparaffinized with xylene and dehydrated through a graded ethanol series. Antigen retrieval was achieved for 20 min in heat-activated antigen retrieval pH 9.0 (Dako, Carpinteria, CA) using a pressure cooker (Dako). Endogenous peroxidase activity was quenched with 3% H_2_O_2_ in water for 15 min. The sections were incubated with mouse monoclonal anti-CD44 antibodies (Dako; Clone DF1485) at 1:100 for 1 h. Subsequently, antigen-antibody reaction was detected with EnVision + Dual Link System-HRP (Dako) and visualized with DAB+ (3, 3′-Diaminobenzidine; Dako). Tissue sections were lightly counterstained with hematoxylin and then examined by light microscopy. Negative controls (substitution of primary antibody with TBS) were run simultaneously. Positive controls included human breast carcinoma for CD44 antibodies.

Immunohistochemically stained sections were digitalized using the NanoZoomer 2.0 HT (Hamamatsu Photonics K.K., Japan) at × 20 objective magnification. The images were analyzed using Visiopharm software v4.5.1.324 (Visiopharm, Horsholm, Denmark). The intensity of staining was categorized as 0, 1+, 2+, and 3+ according to the distribution pattern across the TMA cores. The final histoscore was calculated by multiplying the intensity and percentage of staining resulting in score of 0 to 300 [33]. CD44 expression value was dichotomized (positive vs. negative) with the cut-off value (median).

#### Statistical analysis

Biological validation data presented are the mean ± the standard deviation from three independent experiments unless indicated otherwise. All statistical tests were two-sided. A Student’s t test was used for between-group comparisons. The χ2 test was used to determine whether CD44 expression was associated with clinicopathological characteristics, with protein expression data recoded into binary variables. Statistical analyses were performed using SPSS version 21.0 (SPSS Inc., Chicago, IL). A value of *p* < 0.05 was considered statistically significant.

## Results

### Cohort characteristics

A summary of the clinical (Table 1) and molecular characteristics (Tables 2 and 3) are shown for the cohorts. To assess potential selection bias, we show characteristics for the full cohort of LGGs (124) and GBMs (151), as well as the cohorts selected as the Low MS quartile (69) and the High MS quartile (69).

**Table 2:** Molecular profile of GBMs^a^

	Total GBMs	High MS
Expression subclass		
Classical	39 (25.8%)	11 (15.9%)
Mesenchymal	49 (32.5%)	38 (55.1%)
Neural	26 (17.2%)	10 (14.5%)
Proneural	27 (17.9%)	9 (13%)
G-CIMP	8 (5.3%)	0 (0%)
< NA>	2 (1.3%)	1 (1.4%)
MGMT methylation status		
Methylated	53 (35.1%)	20 (29%)
Unmethylated	66 (43.7%)	31 (44.9%)
< NA>	32 (21.2%)	18 (26.1%)
IDH mutation status		
IDH wt	140 (92.7%)	68 (98.6%)
IDH mut (R132H)	7 (4.6%)	0 (0%)
< NA>	4 (2.6%)	1 (1.4%)

^a^adapted from [9]

**Table 3:** Molecular profile of LGGs^a^

	Total LGGs	Low MS
Cluster of Clusters (CoC)		
COC1	50 (40.3%)	27 (39.1%)
COC2	5 (4%)	0 (0%)
COC3	43 (34.7%)	27 (39.1%)
< NA>	26 (21%)	15 (21.7%)
IDH and 1p/19q codel		
IDH wt	11 (8.9%)	3 (4.3%)
IDH mut	43 (34.7%)	24 (34.8%)
IDH mut, 1p/19q codel	39 (31.5%)	24 (34.8%)
< NA>	31 (25%)	18 (26.1%)
Methylation cluster		
M1	8 (6.5%)	4 (5.8%)
M2	10 (8.1%)	9 (13%)
M3	46 (37.1%)	24 (34.8%)
M4	3 (2.4%)	0 (0%)
M5	31 (25%)	17 (24.6%)
< NA>	26 (21%)	15 (21.7%)
miRNA Cluster		
mi1	25 (20.2%)	14 (20.3%)
mi2	69 (55.6%)	40 (58%)
mi3	2 (1.6%)	0 (0%)
mi4	2 (1.6%)	0 (0%)
<NA>	26 (21%)	15 (21.7%)
RNASeq Cluster		
R1	29 (23.4%)	15 (21.7%)
R2	8 (6.5%)	0 (0%)
R3	12 (9.7%)	8 (11.6%)
R4	33 (26.6%)	19 (27.5%)
< NA>	42 (33.9%)	27 (39.1%)
RPPA Cluster		
P1	32 (25.8%)	18 (26.1%)
P2	26 (21%)	12 (17.4%)
P3	7 (5.6%)	1 (1.4%)
P4	19 (15.3%)	13 (18.8%)
< NA>	42 (33.9%)	25 (36.2%)

^a^adapted from [7]

Clinical data (Table 1) included histological type, age, gender, vital status and overall survival of the patients. LGGs, as expected, have a younger age at diagnosis (mean 41.6 versus 60.8). The MS quartiles have similar age at diagnosis to that of the full cohort of the corresponding type (LGG versus GBM). Overall survival of LGGs is significantly longer than for GBMs (mean of 800 versus 338 days for deceased patients, and only 10.5% versus 64.9% patients were deceased at last time of follow up). The MS does not select towards better/worse overall survival for deceased patients within each grade: Low MS quartile patients’ overall survival is 754 versus 800 days for the full LGG cohort, and High MS quartile patients’ overall survival is 307 versus 338 days for the full GBM cohort. However, the Low MS quartile does select for more living patients (97.1% versus 89.5% for the full LGG cohort), and the High MS quartile for a slightly larger percentage of deceased patients (68.1% versus 64.9% for the full GBM cohort).

The full cohort GBMs display the following Verhaak et al. [6] subtypes (as updated in Brennan et al. [9]) (Table 1B): classical (25.8%), mesenchymal (32.5%), neural (17.2%), proneural (17.9%), and G-CIMP (5.3%). The High MS cohort subtypes are classical (15.9%), mesenchymal (55.1%), neural (14.5%), and proneural (13%), with a larger percentage of samples being mesenchymal, and none of the samples with G-CIMP phenotype.

The molecular characteristics of the LGGs according to Brat et al.’s characterization [27] (Table 1C) are as follows. The full cohort LGGs subtype distribution over Cluster of Clusters (COC) is: COC1 (40.3%), COC2 (4%), COC3 (34.7%), and COC4 (21%). Low MSs are similarly distributed: COC1 (39.1%), COC2 (0%), COC3 (39.1%), and COC4 (21.7%), with the main difference being the lack of samples from COC2.

### Mesenchymal signature profiles of high and low grade gliomas

**Fig. 1:**
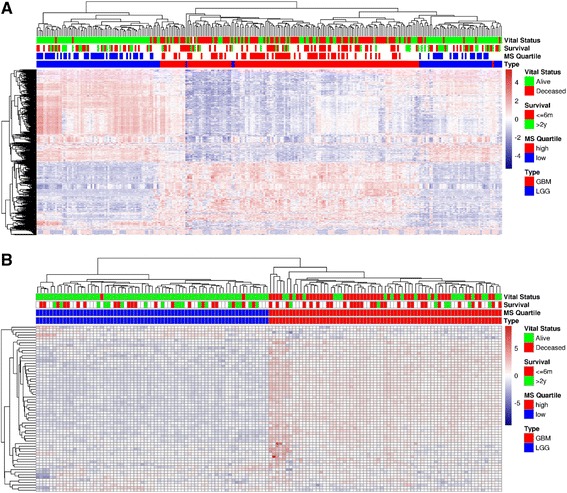
Cohort selection. Unsupervised clustering of gene expressions was used to inspect how LGGs and GBMs clustered. **a** Expressions on the full cohort were clustered according to the 500 genes most varied across samples. **b** Expression of Cheng et al.’s 64-gene was averaged to represent a “metagene”; The (69) samples in the lowest metagene-expression quartile became the “Low MS” cohort, whereas the (69) samples in the highest metagene-expression quartiles became the “High MS” cohort

Mesenchymal phenotype is associated with clinical aggressiveness and stem-like properties in epithelial cancers [17, 34–36]. In GBM, stem-like cells were associated with tumor initiation and treatment resistance [20]. We investigated and confirmed an association between patients expressing high MS signature and poor survival. Cohort selection of patients was performed by stratifying patients (GBM and LGG) by meta gene expression of the mesenchymal transition 64-gene signature shown by Cheng et al. [24]. This gene set was shown to be associated with poor prognosis in GBMs, and to represent biological processes of MS applicable to solid cancers in a multi-cancer setting [23, 24]. Patients stratified by quantile analysis as described in methods, were subjected to hierarchical cluster analysis. High and low MS cohorts cluster into distinct groups (Fig. 1b). In contrast, samples selected by non-specific high variant genes fail to group into clear clusters (Fig. 1a). Patient samples within the low MS cohort consist exclusively of LGG samples and include the majority of the long term survivors (>2 years), while the high MS cohort consists of GBM samples and the majority of short term survivors (< 6 months). The threshold cutoffs of < 6 m and > 2 years for short and long survival have been reported before [37] and are used to show differences between the survivors only in the visualization plots. While the differences in survival are not surprising (since LGGs are the least aggressive among gliomas), we were interested in discovering the regulatory machinery behind the expression of distinct MS signatures, and the biological markers conferring the most prognostic significance.

### Regulatory differences between Low and High MS cohorts

To investigate the differences in regulatory networks between the two phenotypes, we used Glass et al.’s PANDA approach [28]. For a given phenotype, PANDA integrates gene expression, and interaction information (from sequence motif data, and optionally Protein-Protein interaction networks) to construct regulatory networks with edges connecting transcription factors to stipulated gene targets. The edges are weighted in units that reflect the confidence level of the inferred regulatory relationship. We constructed such networks for each of the low and high MS phenotypes, and extracted the subnetworks that are unique to each phenotype - in the sense that a given TF-target relationship is predicted to exist in one of the phenotypes but not the other. These unique subnetworks (summarized in Additional file 2: Figure S1A-B) resulted in 35 TFs uniquely targeting 985 genes in the low MS cohort, and 113 TFs uniquely targeting 987 genes in the high MS cohort. 30 TFs and 65 gene targets are common between the 2 subnetworks.

We used several cross validation features of PANDA, including permutation of gene labels when constructing the regulatory networks in order to compare the constructed networks against a background of randomized networks (Additional file 2: Figure S1C). As expected, permutation of gene labels resulted in a decrease in the size of the networks satisfying the predetermined edge-filtering criteria.

**Fig. 2:**
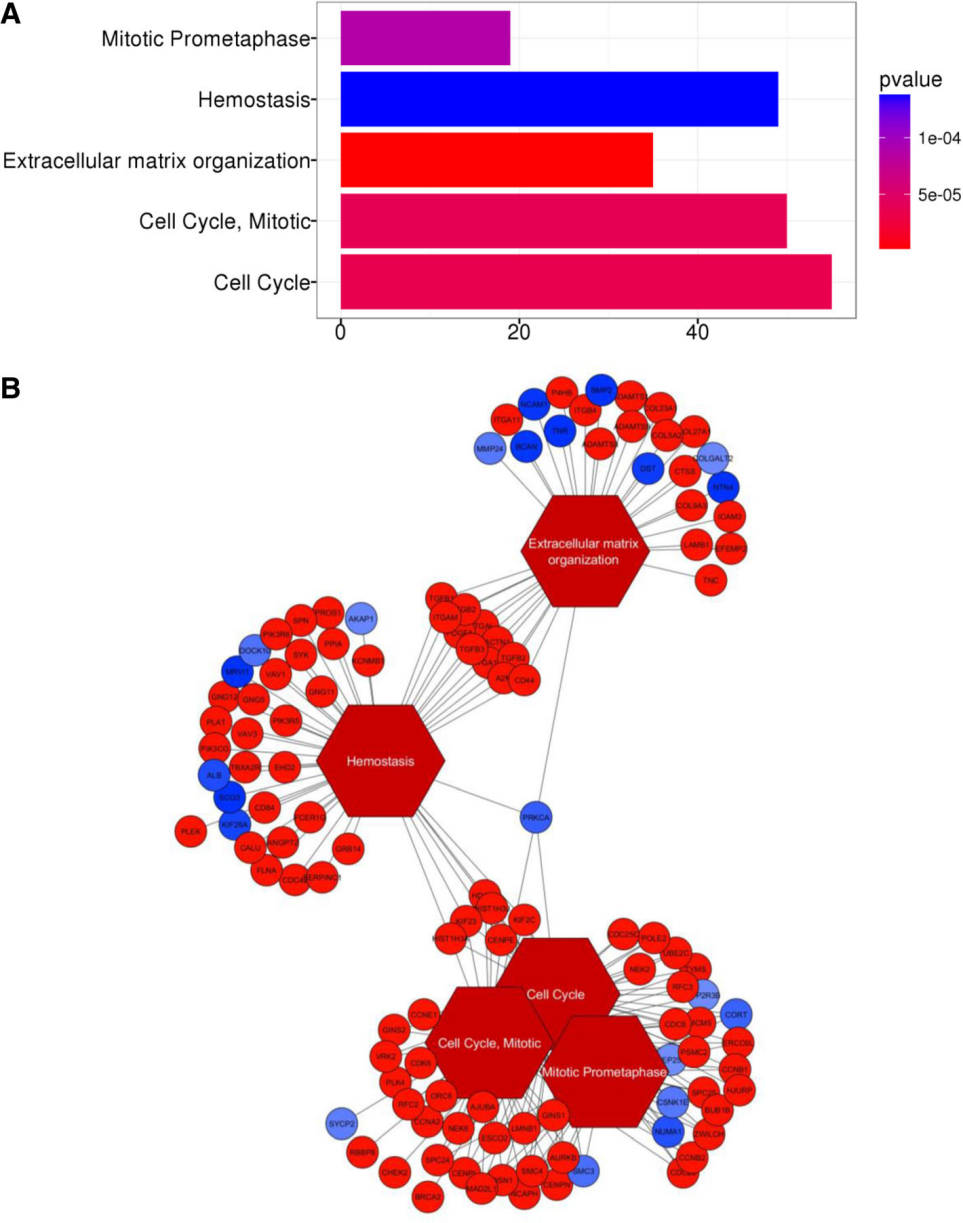
Enrichment of differentially regulated genes. Genes differentially regulated between the regulatory networks for High/Low MS were analyzed for enrichment of Reactome Pathways. **a** Pathways enriched at 0.005 *p*-value of enrichment are shown; Extracellular Matrix Organization is the top enriched category. **b** The enriched pathways with genes colored according to differential expression between High/Low MS phenotypes

PANDA also enables comparison of networks to determine which genes are differentially regulated between the networks, in the sense that the *indegree* - the number of regulatory edges going into a gene -- is significantly different in one of the networks versus the others. one thousand one hundred fifty seven genes were considered differentially regulated between the two networks (with indegree changes *p*-value < = 0.05) and were submitted to pathway enrichment analysis, which is shown in Fig. 2. The top 5-enriched pathways (at *p*-value of enrichment < = 0.005) are shown in Fig. 2a, and the network representation of the pathways is shown in Fig. 2b. The Extracellular Matrix Organization (EMO) pathway is the most significant pathway, and is comprised of 35 genes regulated by 22 TFs according to the network analysis. These 57 EMO genes and TFs were then prioritized by LASSO in a Cox-Proportional Hazards model for prognostic relevance. Covariates of *age*, and *type* (LGG versus GBM) were also included in the model.

**Fig. 3:**
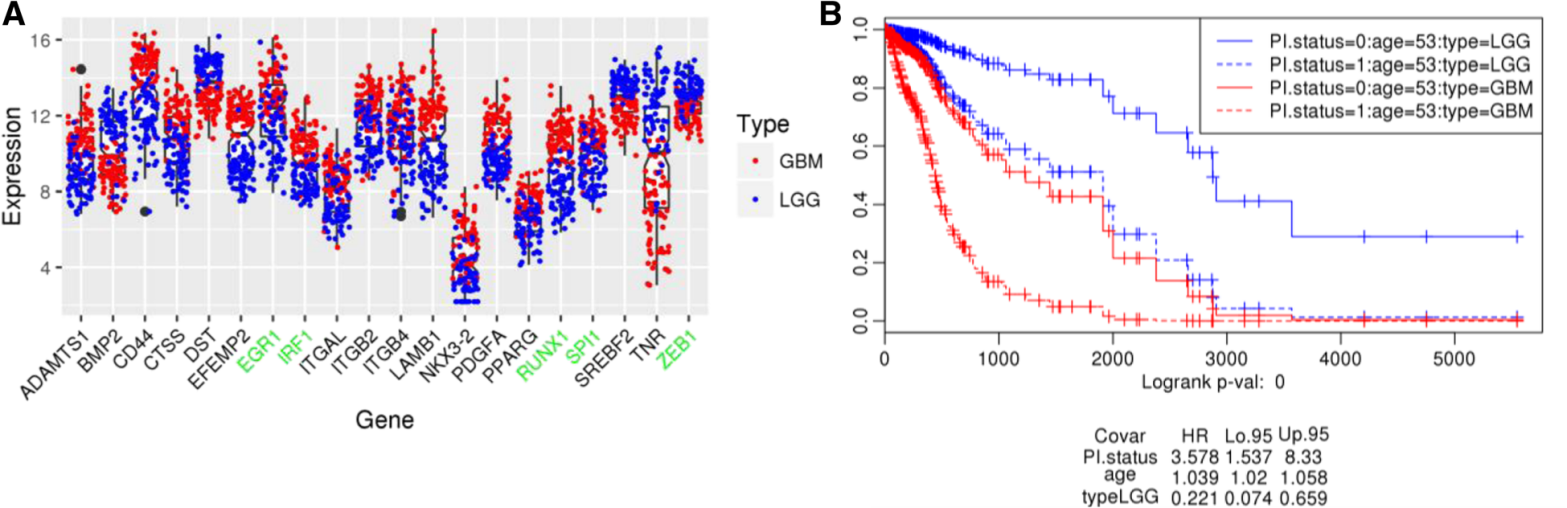
Survival-prioritized EMO genes. EMO genes (and transcription factors regulating them) were prioritized according to their impact on survival. LASSO Penalized Regression was used for Cox-survival prioritization. **a** Expression profiles of 20 genes with non-zero regression coefficients: ADAMTS1, BMP2, CD44, CTSS, DST, EFEMP2, EGR1, IRF1, ITGAL, ITGB2, ITGB4, LAMB1, NKX3-2, PDGFA, PPARG, RUNX1, SPI1, SREBF2, TNR, ZEB1. TFs are labeled in green. **b** Survival analysis based on a multi-gene prognostic index stratification. *Age* and *type* (LGG versus GBM) were also included in the model

The LASSO analysis resulted in selection of a model with significant prognostic value that consisted of 20 genes (with non-zero coefficients), and *age*. As shown in Fig. 3a, 15 genes (ADAMTS1, CD44, CTSS, EFEMP2, EGR1, IRF1, ITGAL, ITGB2, ITGB4, LAMB1, NKX3-2, PDGFA, PPARG, RUNX1, SPI1) were upregulated and 5 genes (BMP2, DST, SREBF2, TNR, ZEB1) downregulated in the high MS cohort (the underlined genes are TFs). Of these, several TFs are known to be involved in the regulation of mesenchymal transition. A Prognostic Index (PI) value was derived using the 20 gene signature, and tested in a Cox regression analysis along with *age*, and *type* (LGG versus GBM) as additional covariates. As shown in Fig. 3b., the impact of the 20-gene associated PI on survival is significant (*PI* HR = 3.578), independently of the other two covariates. As expected, LGGs have a reduced hazard compared to GBMs (LGG *type* HR = 0.221), and age has a small but significant impact on survival (*age* HR = 1.039).

To evaluate the impact of each of the 20 genes on survival, a Cox proportional hazards model was constructed for each gene along with *age* and *type* (LGG versus GBM) as covariates. All models were found to be significant (model *p*-value = 0) (Additional file 3: Figure S2). However, only 9 of the genes had individual prognostic significance: CD44, CTSS, IRF1, ITGB2, NKX3-2, PDGFA, PPARG, SPI1, SREBF2 (the underlined genes are TFs).

### Validation of 20-gene signature in independent data

**Fig. 4:**
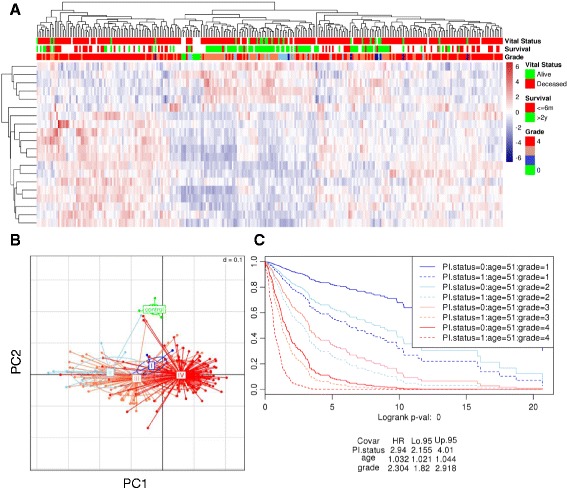
Validation with independent data. GSE16011 data set was used to validate the Lasso-20 signature with respect to impact on survival. The dataset included glioma grades I-IV, and 8 controls. To show the relevance of using all grades, we have included grade III samples in the validation. **a** Unsupervised expression clustering. **b** Principal Component Analysis showing the first two components. Each dot represents a sample plotted against expression levels of 20 genes. Samples are colored according to the grade, green being the normal controls. **c** Cox proportional hazards survival analysis for grades I, II, IV. The model includes *age*, and *grade* as covariates, and the 20-gene Prognostic Index

We validated the prognostic significance of the 20-gene signature in an independent cohort of patient samples, using the publicly available (GEO) microarray data set GSE16011. GSE10611 included 8 normal, 8 Grade 1, 24 Grade 2, 85 Grade 3, and 159 Grade 4 glioma samples, as detailed in Additional file 1: Table S1. We evaluated the discriminatory power of the signature by performing unsupervised hierarchical cluster analysis of the samples based on the LASSO 20 genes (Fig. 4a). We observed three main clusters: the first dominated by Grade 3 and 4 glioma samples, the second dominated by normal samples and samples from Grades 1–3, and a third mixed cluster. PCA analysis is visually intuitive and an alternate way to cluster analysis in which new variables called principal components were identified that capture the highest spread of data from the linear combinations of original variables. Using first 2 components, the analysis further confirmed that the 20-gene signature can distinguish normal and glioma grades (Fig. 4b). To test the combined power of the 20-gene signature, we used the *PI* index, with *age* and *grade* as additional covariates to construct a Cox Proportional Hazards model. The analysis (Fig. 4c) showed that *PI* (HR = 2.94, 95% CI = 2.155–4.01), *grade* (HR = 2.304, 95% CI = 1.82–2.918), and *age* (HR = 1.032, 95% CI = 1.21–1.044) are all significant covariates.

### Validation of CD44 gene as an EMT target

#### CD44 inhibition affects cell proliferation and clonogenic potential in GBM cells

As we initially identified and then validated the 20-gene signature in TCGA and GEO datasets respectively, one of the genes included was gene CD44, a stem cell marker. The survival analysis of LGG and GBM showed that CD44 is an independent prognostic marker (CD44 HR = 1.82, in a model that included *age* and *type*; see Additional file 3: Figure S2). Knowing the role of CD44 in tumor cell migration, we validated this function in GBM tumor cells and stem cells.

**Fig. 5:**
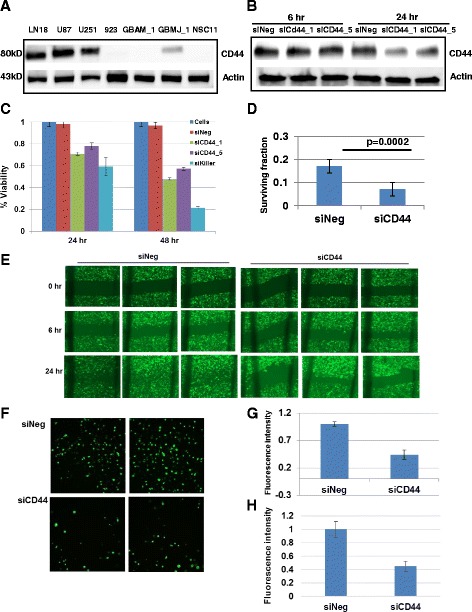
CD44 inhibition is associated with decreased cell viability and migration/invasion in GBM cancer and stem cells. **a** Western blot analysis showing differential expression of CD44 in GBM tumor (LN18, U87, and U251) and stem cells (923, GBAM1, GBMJ1, and NSC11). Analysis of siCD44 transfected U251 cell line showed **b** substantial inhibition of CD44 expression 24 h post transfection using 2 different siRNAs, **c** significant time course reduction of cell viability, and (**d**) inhibition of long term survival using clonogenic assay. **e** A representative wound healing assay showing diminished migration of U251 cells in siCD44 transfected cells compared to control. **f**-**h**. Boyden chamber assay showing F. decreased ability of U251 cells transfected with CD44siRNA through the matrigel coated membrane compared to control. Chemoattractant was 1% FBS. Quantified data depicted in (**g**). and bars represent the mean of 3 independent experiments measured in triplicate +/- standard deviation (**h**). Stem cells shown to have minimal CD44 expression had a decreased ability to migrate towards U251 cells compared to negative transfected control. Chemoattractants were FBS and stem cell growth factors (bFGF and EGF)

First we examined CD44 expression in a number of GBM tumor cells and stem cells (Fig. 5a). GBM tumor cells expressed CD44 whereas the GBM stem cells tested had minimal to no CD44 expression. Next, to study the effect of CD44 on GBM tumor cell phenotype, using siRNA methodology, CD44 expression was downregulated in U251 tumor cells with two different siRNAs. We observed a substantial decrease in CD44 protein levels, 24 h post-transfection (Fig. 5b). Moreover, the decrease in CD44 levels, was associated with a significant reduction in the viability of the GBM cell line U251 (Fig. 5c). Further, downregulation of CD44 in GBM cells, inhibited its long term clonogenic survival ability (Fig. 5d). These results indicate that, CD44 expression could be necessary for GBM cell survival.

#### CD44 plays an important role in cell migration and invasion

Next we confirmed role of CD44 in two EMT specific characteristics; tumor cell migration and invasion. Migration was assessed by a scratch assay in U251 GBM cells transfected with siNeg/siCD44 (Fig. 5e). Migration of individual cells in the leading edge of the scratch was followed for 24 h. At 24 h most of the wounds had closed in U251 cells transfected with siNeg. However, cells transfected with siCD44 migrated at a slower rate in the leading edge of the scratch.

To confirm further, we used Boyden chamber assay to assess the invasive properties of GBM U251 tumor cells. This in vitro test allows the rapid and quantitative assessment of invasiveness. Using a chemoattractant (1% FBS), we observed that inhibition of CD44 expression abrogated the tumor cell ability to invade through a matrigel coated membrane (Fig. 5f and g). Representative images of U251 migration through the madrigel coated membrane are shown in Fig. 5f with bar plots showing quantitative data (Fig. 5g).

As we observed minimal to no expression of CD44 in our stem cell lines (Fig. 5a), we wanted to examine if GBM tumor cells mediate stem cell migration. To test that we examined invasion of NSC11 GBM stem cells towards the tumor cells. This was achieved by plating stem cells on the top chamber and measuring their migration/invasion in the lower chamber in the presence of U251 tumor cells (Fig. 5h). U251 cells transfected with siNeg. control, attracted a sufficient number of stem cells across the membrane. However, U251 cells with downregulated CD44 expression attracted significantly less stem cells across the matrigel coated membrane (Fig. 5g).

#### CD44 is overexpressed in GBM tissues

**Fig. 6:**
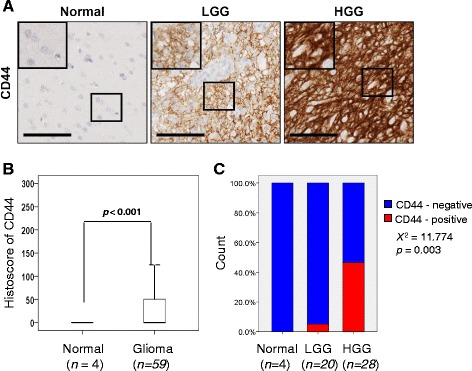
CD44 expression in brain tumor tissues. **a** Representative images of immunohistochemical staining of CD44 in brain tissues from normal, low grade glioma (LGG), and high grade glioma (HGG). The boxed regions are displayed at high magnification in the inset (scale bar: 100 *μ*m). **b** Box plot depiction of immunohistochemical staining data. The histoscores were computed based on intensity and tissue area of positive staining. Error bar represents mean ± s.d. **c** High CD44 expression was strongly associated with advanced stage of brain tumors. We analyzed only 58 cases with available grade information. LGG, low grade glioma (grade I + II); HGG, high grade glioma (grade IV)

Next, we assess the levels of CD44 expression in patient clinical samples. The level and pattern of CD44 expression in normal brain tissues and brain tumor tissues was examined by immunohistochemical staining. CD44 expression was primarily observed in the membrane of glioblastoma cells. Representative immunohistochemical staining images of CD44 are presented in Fig. 6a. Of 59 gliomas, 24 (40.7%) were categorized as positive membrane staining. When compared with normal brain tissues, CD44 expression was significantly overexpressed in brain tumor tissues (Fig. 6b, *p* < 0.001). Furthermore, CD44 expression was significantly associated with the glioma grade; 5% in LGG versus 46% in GBMs (Fig. 6c).

## Discussion

The seminal feature of gliomas is their diffuse infiltrative nature, which renders them incurable by the existing standard of care. Infiltrating glioma cells exist in close proximity with components of the tumor microenvironment, including extracellular matrix components in the brain. GBM cells undergo a series of molecular and conformational changes shifting the tumor toward mesenchymal traits, including extracellular matrix remodeling, cytoskeletal re-patterning, and stem-like trait acquisition [38]. EMT is considered a major modulator of metastasis in epithelial solid tumors; EMT(-like) processes have been reported in the case of tumors of neuroepithelial origin [39]. A deeper understanding of the mechanisms driving and regulating infiltration represents the first step toward successful treatment of this pathology. In the current study, we address this issue by integrating publically available data using a number of computational approaches in search of a prognostic index gene signature for gliomas. Identification of predictive markers of survival in gliomas could optimize and individualize therapy by prospectively identifying the patients that will benefit the most from standard therapy, and identifying novel therapeutic targets based on the molecular profiles of the patients that are refractory to standard therapy.

Studies have demonstrated that bioinformatics approaches in general, and systems biology in particular, are powerful in identifying metastasis specific gene signatures, predicting disease outcome, and elucidating mechanisms of cancer progression [35]. In the current study, we used these approaches to elucidate differentially regulated genes and transcription factors in low grade and high grade gliomas. We started out with a hypothesis that the high MS cohort is characterized by distinct gene expression patterns compared to the low MS cohort. We used the 64-gene mesenchymal transition metagene signature [24] to stratify patients into high and low MS cohorts, and looked for additional functions in these cohorts related to long term survival. We performed regulatory network and pathway analyses. Not-surprisingly, we found that the most differentially regulated pathways were related to the extracellular matrix organization (EMO). To evaluate the prognostic relevance of EMO we prioritized the EMO associated genes and TFs using LASSO with Cox regression option, which resulted in a 20 gene set (15 genes and 5 TFs). The TFs include ZEB1, EGR1, IRF1, RUNX1, and SPI1. Several genes of this subset were found to be individually associated with cancer. RUNX1 has been identified as a key regulator of tumorigenesis in various epithelial cancers [34] including breast [40] and lung cancers [41]. IRF1 has been implicated in bevacizumab-resistant tumors [42]. EGR1 has important functions in the regulation of growth and differentiation, and is highly expressed in brain. It was demonstrated that EGR1 positively regulates the activity of the FN gene, and that cell adhesion and migration were greatly increased in the EGR1-expressing glioblastoma cells [43]. ZEB1, a zinc-finger protein, is an inducer of EMT, through downregulation of E-cadherin and upregulation of vimentin [44]. It is also a transcriptional repressor of cell-adhesion genes and several microRNAs, particularly members of miR-200 family, which function not only as strong inducers of mesenchymal-epithelial transition (MET) but also inhibit undifferentiated stem cell properties [45]. However, in our cohort, ZEB1 was overexpressed in LGGs, as compared to HGGs. The Cox model with ZEB1, *age*, and *type* as covariates showed no significant differences in survival based on the ZEB1 expression alone. We explored the relationship of ZEB1 expression and survival for each GBM subtype using GBM-BioDP [32] and found that for the proneural GBM, higher expressions of ZEB1 are associated with better prognosis.

Our cohort selection was initially based on a 64 gene signature. Our focus was on regulatory differences between the two cohorts, and additional functions associated with survival length, resulting in a 20 gene signature with no overlap with the original 64 gene signature. A possible reason for the lack of overlap is that Chen et al tested for “time to recurrence” [24]. Recurrence is the biological outcome of many processes, with MS being only one of them. Our 20-gene signature can be used as a better classifier of the MS process than the 64-gene signature. Moreover, Cheng and co-workers found that the 64 gene signature is strongly correlated in gliomas with the putative stem cell marker CD44, and is highly enriched among the differentially expressed genes in glioblastomas vs. lower grade gliomas. This finding correlates with our identification of CD44 gene being one of the targets in our study population.

CD44 gene has been shown to play a significant role in the EMT phenotype in various cancers. It is a complex transmembrane glycoprotein that serves as a receptor for the extracellular component hyalouronic acid. CD44 expression was implicated to be elevated in tumor-initiating cells in many kinds of cancers [46]. Thus, CD44 is thought to be a biomarker for cancer stem cells (CSCs) [47]. Functional studies have shown that CD44 is involved in tumorigenesis and metastasis in many cancer types such as colon [48–50], bladder [51], gastric [52], breast, [53], and GBM [54]. Recently, overexpression of CD44 was associated with significantly worse overall survival in breast cancer patients [55]. Given the role of CD44 in human cancers, we validated our computational modeling results in various experimental settings. We measured CD44, expression levels in low grade gliomas (grade1 and 2) and high (grade 44) GBM. Our analysis revealed a strong association of CD44 overexpression with clinicopathological features, including the histological grade in GBM patient samples. Downregulation of CD44 expression decreased GBM cell proliferation and their invasion and migration properties. Downregulation of CD44 also inhibited the GBM cells’ long term clonogenic survival ability. GBM tumor cells expressing CD44 could attract stem cells, however inhibition of CD44 expression abrogated tumor cell mediated chemotactic activity towards the stem cells. This is important, as stem cells in tumor niches are responsible for chemo- and -radioresistance. As a proof of principle, the results reported here indicate the validity of our computational modeling in predicting the prognostic index for high grade gliomas.

The biomarkers were validated using an independent dataset. The 20-gene signature not only separated LGG and HGG, but also the normal and various glioma grades from each other. Thus the additional validation of the signature in independent patient samples suggests the robustness of the signature in identifying patients transitioning to aggressive mesenchymal phenotype. Our finding that the 20-gene expression could discriminate long term vs. short term survivors raises the possibility of using their expression signature to develop rapid and accurate molecular diagnostic test to predict survival length in gliomas. Furthermore, our integrated model provides us with new insights into the molecular determinants of mesenchymal transition kinetics in GBM. Functional characterization of this signature in in-vivo experiments with cell lines and mouse models will shed light on their biological importance in GBM progression. Further development of computational tools could bridge one of the critical missing links between in vitro drug screening and in vivo drug activity.

## Conclusion

Our study ﻿on computational analysis of mesenchymal signature (MS) in gliomas reveals a differential expression of gene networks and transcription factors between low grade and high grade gliomas. We identified a core group of candidate MS driver genes over-expressed in high grade gliomas and shown to be strongly associated with poor survival. This computational network based strategy of identifying MS signature genes is validated in an independent data set and potentially, a valuable tool to identify functional or mechanistic gene differences in cancer. Ultimately this has the potential to transform our understanding of how the grade IV glioblastomas are formed and maintained, a question that is fundamental to GBM biology.

## Additional files

Additional file 1: Table showing the summary of clinical characteristics of independent data (XLSX 9 kb)
Additional file 2: Figure S1. showing the PANDA differential expression results of gene and transcription factors between LGG and GBM. Specificity of network edge weight and FDR were compared after randomization of gene labels. (PPTX 1390 kb)
Additional file 3: Kaplan Meier plots of 20 genes selected by LASSO based gene prioritization, showing the prognostic relevance of each gene in LGG and GBM. (PPTX 1298 kb)

## Abbreviations

CI: Confidence Interval
COC: Cluster of Clusters
EMO: Extracellular Matrix Organization
EMT: Epithelial to Mesenchymal Transition
FDR: False Discovery Rate
GBM: Glioblastoma
GEO: Gene Expression Omnibus
HGG: High Grade Glioma
HR: Hazard Ratio
LGG: Low Grade Glioma
MS: Mesenchymal Signature
PANDA: Passing messages between biological networks to refine predicted interactions
PCA: Principal Component Analysis
PI: Prognostic Index
PPI: Protein-Protein Interactions
TCGA: The Cancer Genome Atlas
TF: Transcription Factor
TMA: Tissue Microarray
