# Supplementary material for: Computational analysis of the mesenchymal signature landscape in gliomas
Source: BMC Med Genomics. 2017 Mar 9;10:13. doi: 10.1186/s12920-017-0252-7 (PMC5345226; doi:10.1186/s12920-017-0252-7)
Supplement: Additional file 2: Figure S1. — showing the PANDA differential expression results of gene and transcription factors between LGG and GBM. Specificity of network edge weight and FDR were compared after randomization of gene labels. (PPTX 1390 kb) [file 12920_2017_252_MOESM2_ESM.pptx]

## Slide 1
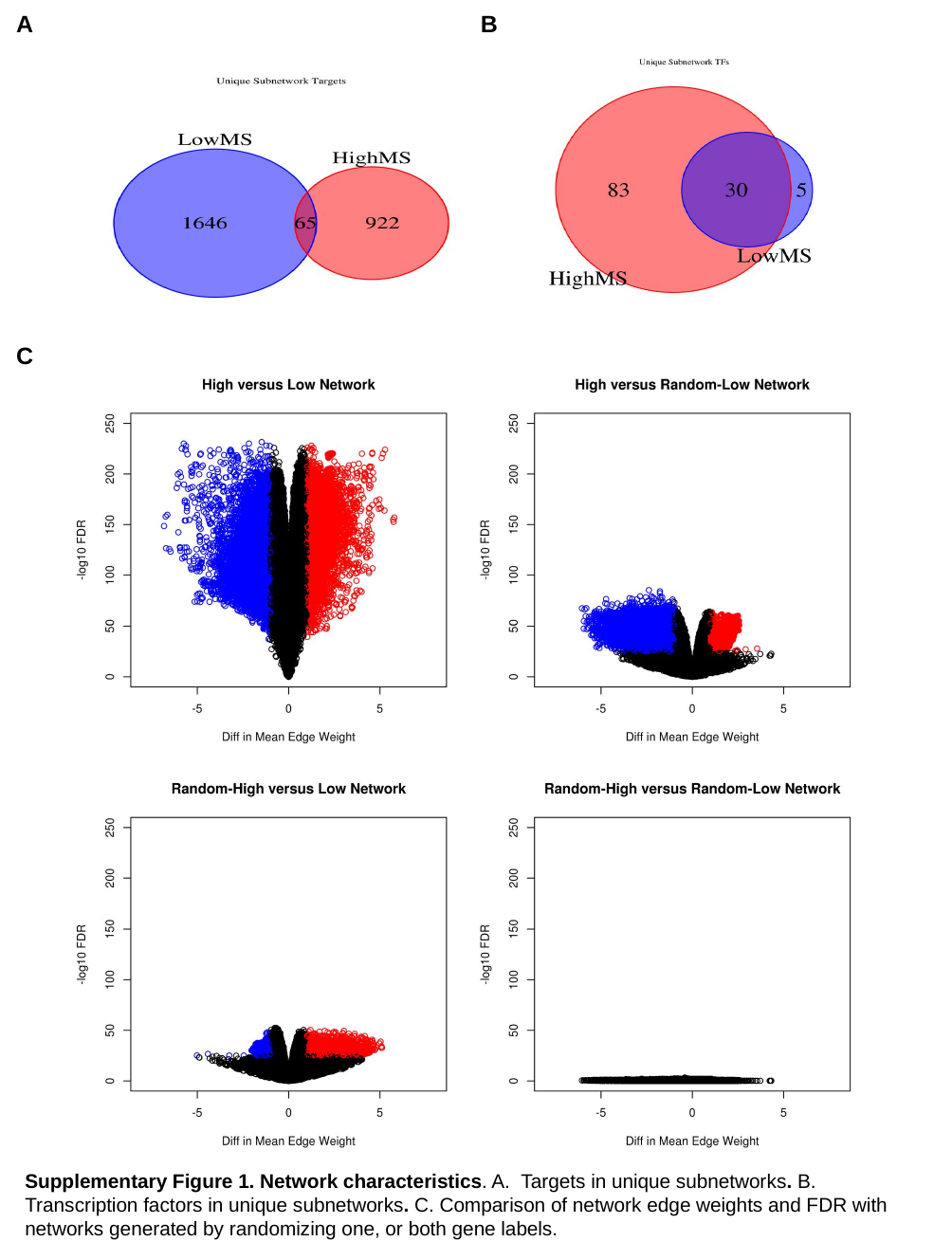

B
A
C
Supplementary Figure 1. Network characteristics. A. Targets in unique subnetworks. B. Transcription factors in unique subnetworks. C. Comparison of network edge weights and FDR with networks generated by randomizing one, or both gene labels.
