# Supplementary material for: Computational analysis of the mesenchymal signature landscape in gliomas
Source: BMC Med Genomics. 2017 Mar 9;10:13. doi: 10.1186/s12920-017-0252-7 (PMC5345226; doi:10.1186/s12920-017-0252-7)
Supplement: Additional file 3: — Kaplan Meier plots of 20 genes selected by LASSO based gene prioritization, showing the prognostic relevance of each gene in LGG and GBM. (PPTX 1298 kb) [file 12920_2017_252_MOESM3_ESM.pptx]

## Slide 1
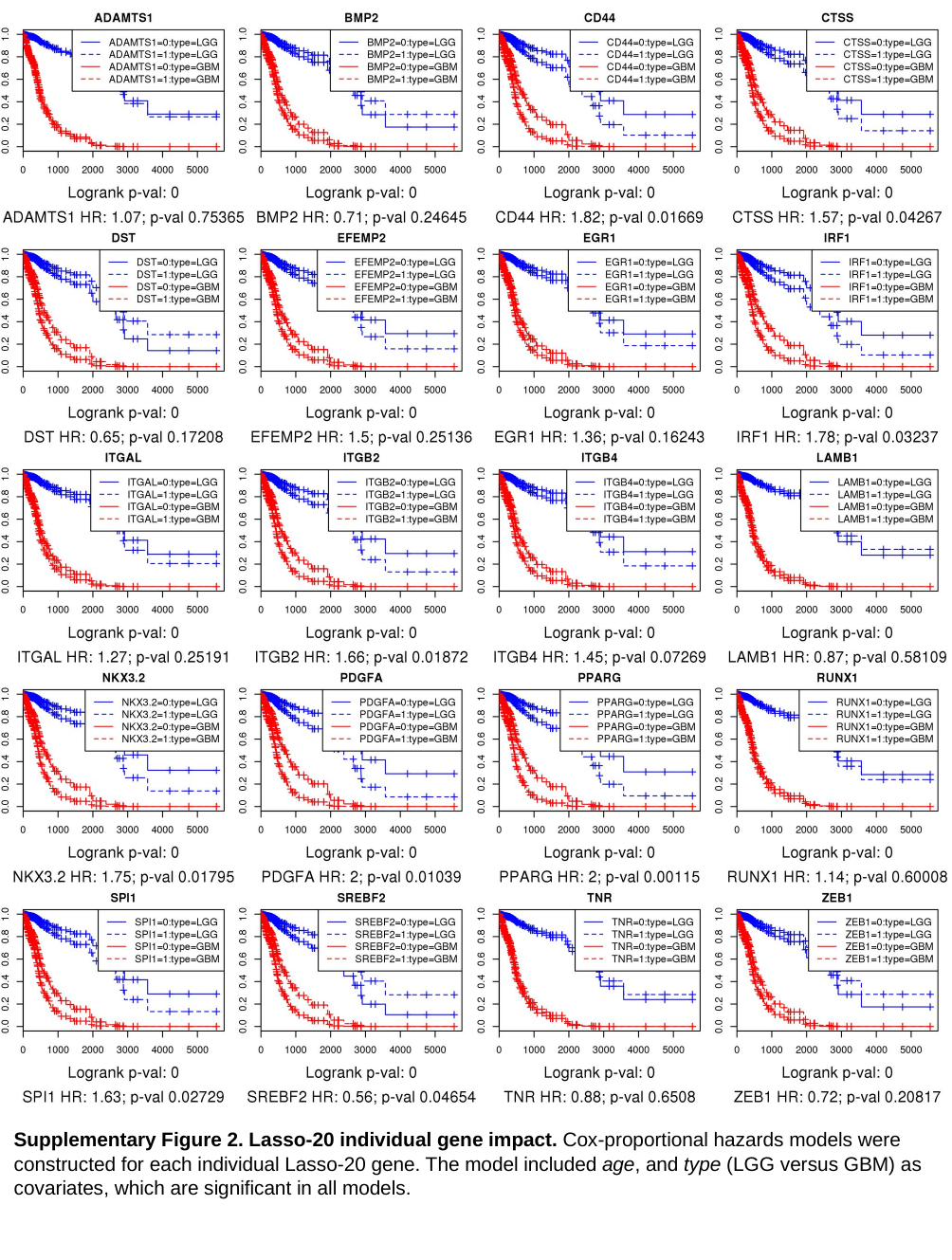

Supplementary Figure 2. Lasso-20 individual gene impact. Cox-proportional hazards models were constructed for each individual Lasso-20 gene. The model included age, and type (LGG versus GBM) as covariates, which are significant in all models.
